# TASOW – A tool for the automated selection of potential windbreaks

**DOI:** 10.1016/j.mex.2022.101826

**Published:** 2022-08-24

**Authors:** Simon Scheper, Barbara Kitzler, Thomas Weninger, Peter Strauss, Kerstin Michel

**Affiliations:** a Dr. Simon Scheper – Research | Consulting | Teaching, DE-29413 Dähre, Germany; b Federal Research and Training Centre for Forests, Natural Hazards and Landscape, AT-1131 Vienna, Austria; c Environmental Geosciences, University of Basel, CH-4056 Basel, Switzerland; d Federal Agency for Water Management, Institute for Land and Water Management Research, AT-3252 Petzenkirchen, Austria

**Keywords:** TASOW, Tool for Automated selection of Windbreaks, RWEQ, Revised Wind Erosion Equation, Wind erosion, Risk, Soil protection, Ecosystem service, Land use, RWEQ

## Abstract

Wind erosion is a process in which soil particles are detached from soils and transported downwind. One effective measure to reduce wind erosion are vegetated windbreaks such as hedgerows as they reduce wind speeds and likewise the forces which detach and transport soil particles.

However, the planting of new windbreaks is driven by policy decisions as well as planning considerations. To get an initial idea of potential locations for new windbreaks, we present an automated routine as a model in ESRI ArcGIS Pro to propose plantation locations. The main input to the model is a wind erosion risk map. The results are potential locations for windbreaks that are ranked according to their suitability. The model parameters are adjustable, transferable to other regions and can be altered by to the user's needs.

• Limit the wind erosion risk map to the most prone fields
• Selection of unprotected sites perpendicular to the main wind direction
• Suggestions for suitable sites for the potential planting of new windbreaks

## Highlights

• Limit the wind erosion risk map to the most prone fields.
• Selection of unprotected sites perpendicular to the main wind direction.
• Suggestions for suitable sites for the potential planting of new windbreaks.

## Graphical abstract

**Figure:**
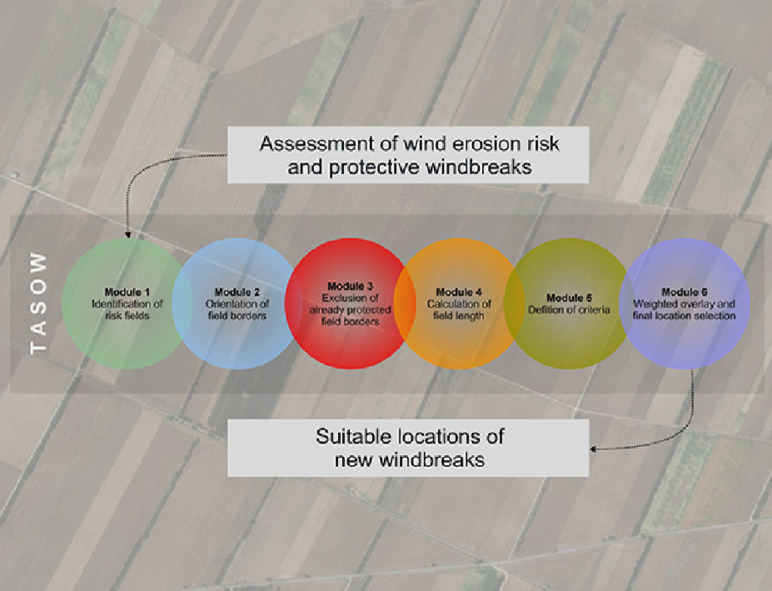

Specifications Table

**Table:** 

Subject Area:	Environmental Science
More specific subject area:	Land use planning
Name of your method:	**T**ool for **A**utomated **s**election **o**f **W**indbreaks (TASOW)
Name and reference of original method:	N.A.
Resource availability:	ESRI ArcGIS Pro 2.9 [1]

**Method details**

## Aim of the method

Amongst other possibilities, vegetated windbreaks are widely applied as measures to significantly reduce wind speeds and thus soil erosion rates in regions where vital tree growth is possible [2]. Previous results demonstrated that the agricultural fields in the eastern surroundings of Vienna, Austria, have a mean modeled soil loss rate of 3.7 t ha^−1^ yr^−1^ by wind erosion [3]. Although the study region is known for its windbreaks that have been planted since the 1950s [4], many agricultural fields are unprotected by wind-breaking obstacles and thus at risk.

To determine locations for a potential planting of windbreaks, we developed an automated procedure (**T**ool for **A**utomated **s**election **o**f **W**indbreaks, TASOW) that can help policy makers to decide whether additional vegetated windbreaks are appropriate and where to place them. This automated routine was developed in ESRI ArcGIS Pro 2.9. and can be applied or adapted to wind erosion modeling results different from those of the authors and the demonstration region. Herein, the model structure of TASOW is described in detail and its application is demonstrated in a case study for an agriculturally dominated area in eastern Austria.

## Methodological basics

The model is designed as a multistep model which fulfills different requirements and takes assumptions to finally select potential locations of windbreaks. The theoretical background of the presented method is the areal estimation of wind erosion risk. The TASOW model was developed using the Revised Wind Erosion Equation (RWEQ, [5]), which is a widely used model for predicting potential soil loss rates and wind erosion susceptibility in agricultural fields [6]. The RWEQ is based on physical and empirical considerations, was originally developed for field scale predictions, and is nevertheless frequently used in spatial GIS-based studies (e.g. [3,7,8]) .

Basically, the method is based on the assumptions that the wind protection effect of newly planted windbreaks may be maximized by locating these structures where i) a high wind erosion risk is estimated, ii) no present windbreaks exist, iii) field orientation is in the main wind direction, and iv) field length is high. The assumptions can be changed by the user according to the needs of the respective study area and the used wind erosion risk model. In addition, windbreaks play a crucial role for many other ecosystem services like water balance, cultural ecosystem services or biomass production [9] which are not covered in this method.

The first selection of potential windbreak locations is further condensed by a ranking according to three criteria: i) location of potential windbreak at land use units (e.g. farmland, meadow, etc.) eligible for redesign to windbreaks, ii) vicinity to roads to mark the perimeter of field blocks (coherent parceled fields) and also protect roads from external damage by wind erosion, and iii) elements that are long and therefore protect a large portion of a field border. As an output, TASOW presents polygons which represent the locations in the study area where installations of new windbreaks are reducing the wind erosion risk most effectively.

## Input data

Some datasets are required for the automated process to run and identify potential sites for new windbreaks. The base for the routine is a wind erosion risk map (preferably modeled by RWEQ) that contains either soil loss rates or qualitative wind erosion classes. Furthermore, a dataset of the fields within the study area, the extent of the study area, and information on existing windbreaks are required. The main wind direction of the study region must be known.

To establish the criteria, it is recommended to use OpenStreetMap data [10] on roads and land use. Such data can be downloaded free of charge for any country from https://download.geofabrik.de/.

Additional information to validate the results can be e.g. high resolution orthophotos. Table 1 describes the needed datasets and gives references to the actually used data in the demonstrative case study.

**Table 1:** Datasets used for the setup of the automated routine.

Data	Description	Data type	Source in case study
Wind erosion risk map	Soil erosion rates modeled by RWEQ	Rasterized	[3]
Field cadaster	Cadaster map with fields	Vectorized	[11]
Study extent	Extent of the study area	Vectorized	[12]
Windbreaks	Existing windbreaks in the study area	Vectorized	Lower Austrian Authority for Land Reform; manually digitized from orthophotos
Roads	Road network within Austria based on OpenStreetMap	Vectorized	[13]
Land use	Land use categories within Austria based on OpenStreetMap	Vectorized	[13]
Orthophoto	Orthophoto of Austria	Rasterized	[14]
Background information	Basemap, administrative borders	Vectorized, WMTS	[12,15]

## Simplified overview of the method

TASOW first identifies risk fields of soil erosion by water by selecting fields above a certain threshold. Then, the method focuses on the aspect of field borders as only such field borders are relevant within the framework of the model that are perpendicular to the main wind direction. Such fields that are already protected by windbreaks are excluded in a next step. So, this step substitutes a barrier factor (percent of upwind velocity PUV) in the input wind erosion model RWEQ [5]. Fields with a high field length are more relevant to be protected compared to small fields with a low field length. As such, field length is calculated and considered for a selection of specific fields. The user can assign criteria that are suitable for a potential installation of windbreaks. Such criteria could be the proximity to roads or specific land use types that could be converted. Finally, the method results in a proposal of locations that are suitable for new windbreaks in consideration of the defined thresholds and criteria. Please see the graphical abstract for a scheme of the methods framework. The detailed method workflow is attached as supplement material and can be used as model within the ModelBuilder framework or as python script in ESRI ArcGIS Pro.

The single modules are introduced in detail in the next chapter.

## Modules of the automated routine

### Module 1: identification of risk fields

**Fig. 1:**
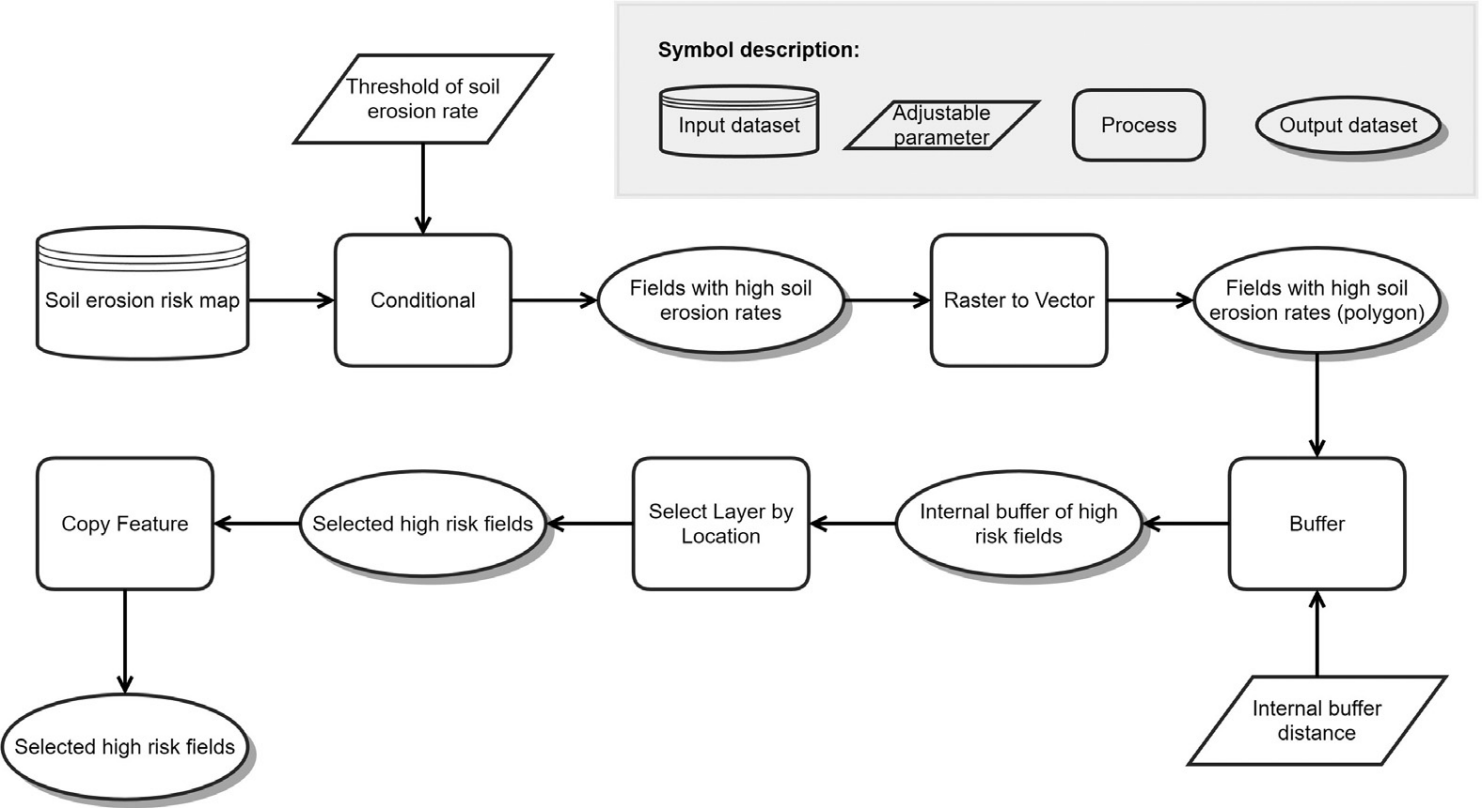
Model structure of module 1 which identifies the fields that are above a chosen threshold (case study: 80^th^-percentile) of mean annual soil erosion loss rates by wind (“high risk fields”) within the study area.

The rasterized input wind erosion risk map is used to identify the fields with highest wind erosion risk in a first step (named “high risk fields” throughout the rest of the paper). Therefore, a threshold needs to be defined above which a field is considered a high risk field. For maps with potential soil loss rates, a quantile should be used; for maps with wind erosion risk classes, a selection of classes needs to be chosen. In a further step, the raster-based fields are used to specify the corresponding vector-based fields in their original extent and shape without any distortion due to the raster size. The high risk fields are converted to vector format and internally buffered by a buffer distance very close to the cell size of erosion risk model to avoid any overlapping in the subsequent location-based selection. The final step results in the vector-based selection of the high-risk fields (Fig. 1).

In the study area, a mean annual wind erosion soil loss rate was calculated using the RWEQ model. To consider only fields with high wind erosion risk, the 80^th^-percentile of all fields in the study area was chosen as a threshold. This percentile can be changed by the user. In our case, the used specific threshold to separate the upper 20% was 0.66 t ha^−1^ yr^−1^. As our input data has a spatial resultion of 10 m, we buffered internally by -9 m. After vectorizing and buffering as described above, a map of high risk fields (Fig. S1) is passed on to module 2.

### Module 2: orientation of field borders

**Fig. 2:**
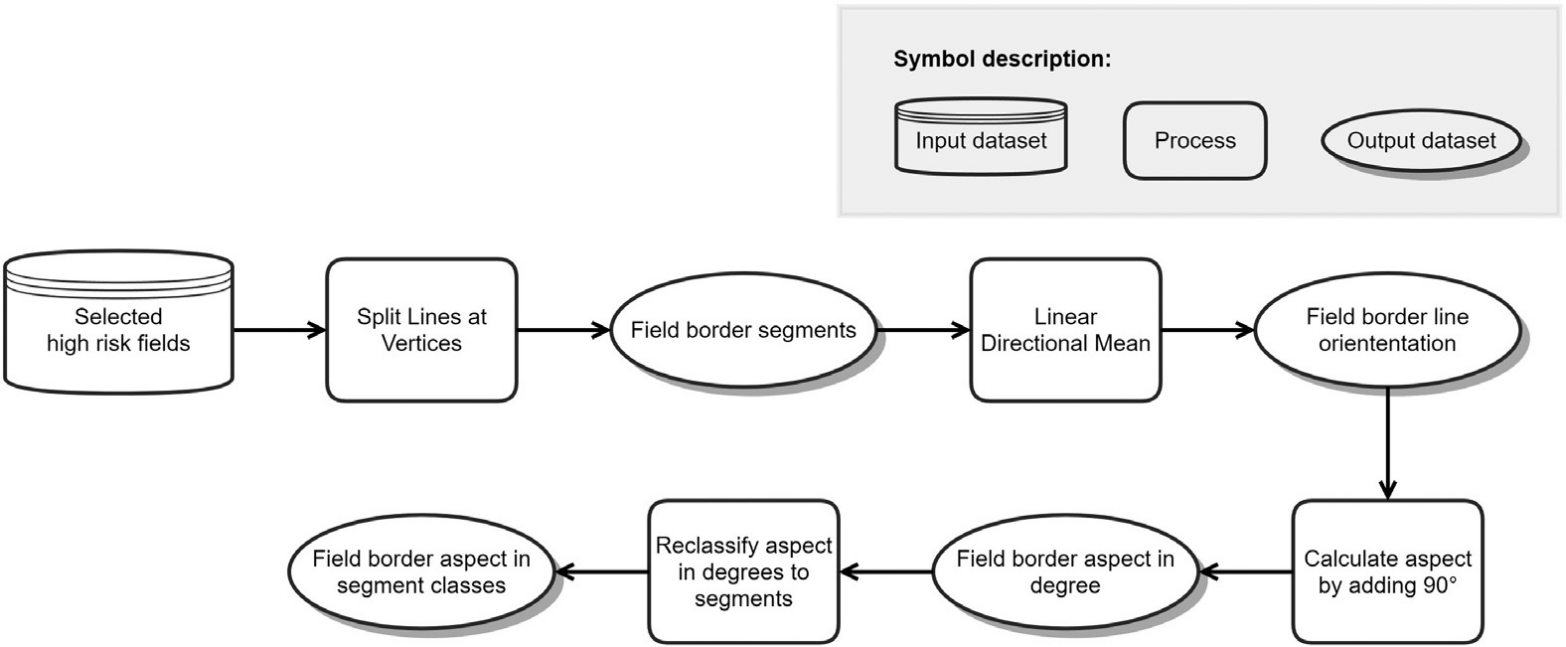
Model structure of module 2 to identify the wind direction that is effective for each field border segment.

In regions where a pronounced main wind direction exists, windbreaks are most effective when oriented perpendicular to this direction. To identify field borders that are perpendicular to the main wind direction, the polygons of the fields are to be decided into lines associated with their orientation. Module 2 (Fig. 2) splits each field polygon into line segments at vertices. The linear directional mean tool then calculates the direction of each line segment. However, since we are interested in a wind direction that is perpendicular to the orientation of the field border, hence the aspect, we need to add 90° to each of the segment directions. This means, if a field boundary line segment is orientated Northwest (315°), the method adds 90° to it to get an aspect of Northeast (45°). In this example, the field border is affected by winds from Southwest (225°) or Northeast (45°). The result of the module (Fig. S2) is a vector file containing all field borders attributed by their aspect. The orientations are aggregated into a total of eight classes (North, Northeast, East, Southeast, South, Southwest, West, Northwest).

### Module 3: exclusion of already protected field borders

If windbreaks are not considered in the integrated wind erosion risk modeling, existing windbreaks need to be excluded at this point as they do not define potential locations for further planting and already serve as protective features for the fields. A dataset which includes such features as complete as possible is needed for the study area to apply this module.

**Fig. 3:**
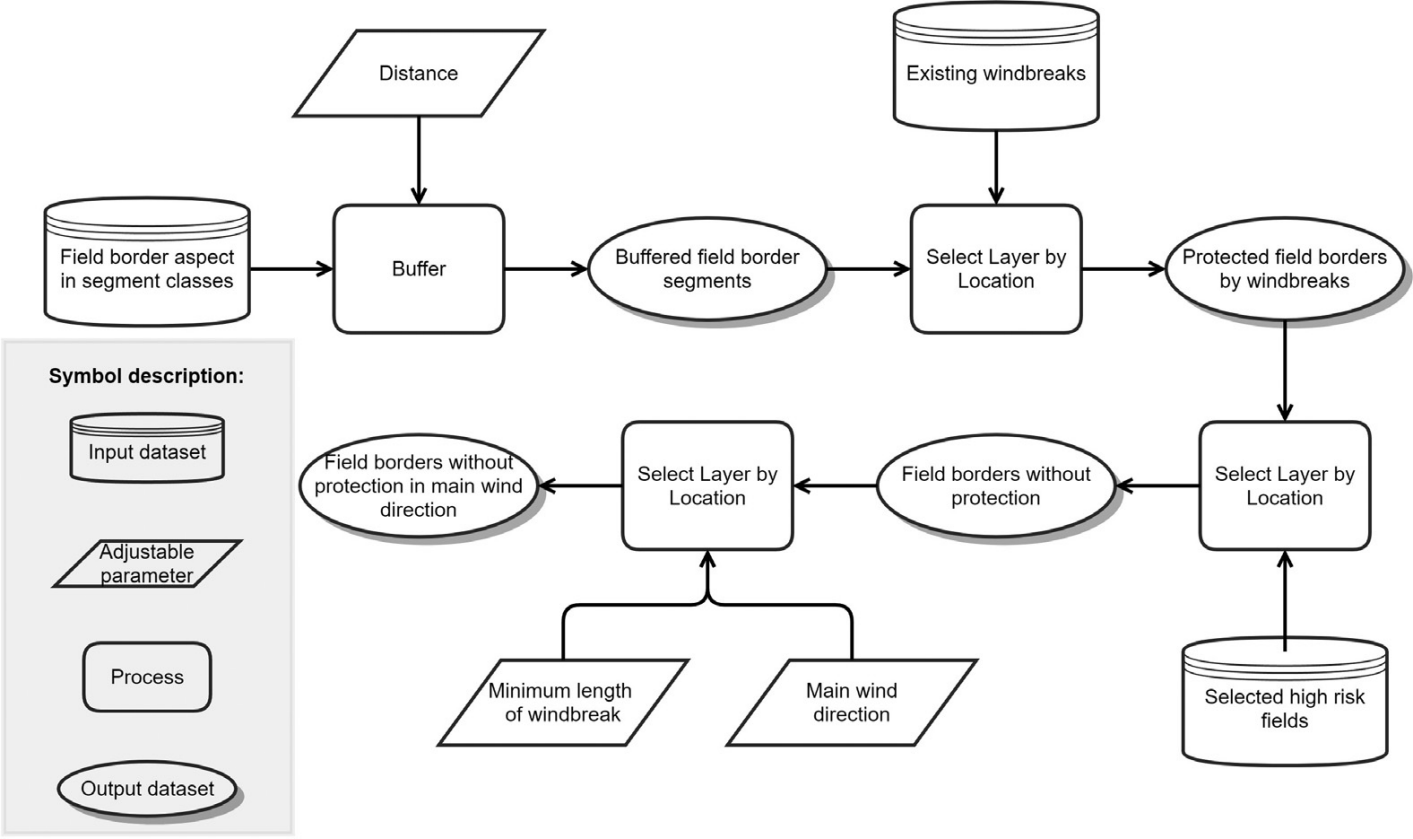
Model structure of module 3 to select only such field borders that are unprotected and perpendicular to the main wind directions.

To start with, the line segments of the field borders provided by the previous module are buffered to enlarge its extent and therefore enable a subsequent location-based selection of already installed windbreaks it its vicinity through an intersection as we assume that windbreaks are not necessarily located exactly at field borders but within a certain distance (Fig. 3). This distance needs to be chosen by the user. The selected field borders that are already protected by existing windbreaks are subtracted from all field borders, remaining in a vector dataset including only field borders that are unprotected. Subsequently, only windbreaks which have an aspect perpendicular to the main wind direction and which have a specific minimal length are considered to avoid too small segments that do not represent real field borders and are just artifacts of the editing process.

In the demonstration area, we define windbreaks as linear wooded obstacles like hedgerows, close tree rows as well as areal obstacles like forests of a minimal length of 30 m. A layer of existing windbreaks was provided by the Lower Austrian Authority of Land Reform and manually completed by editing orthophotos. A buffer distance to define the potential vicinity of field borders to windbreaks of 10 m was chosen and as the main wind direction in the study region is Northwest (315°) and Southeast (135°) [2,3], we selected only field borders that have an aspect of Northwest and/or Southeast, respectively (Fig. S3).

### Module 4: calculation of field length

**Fig. 4:**
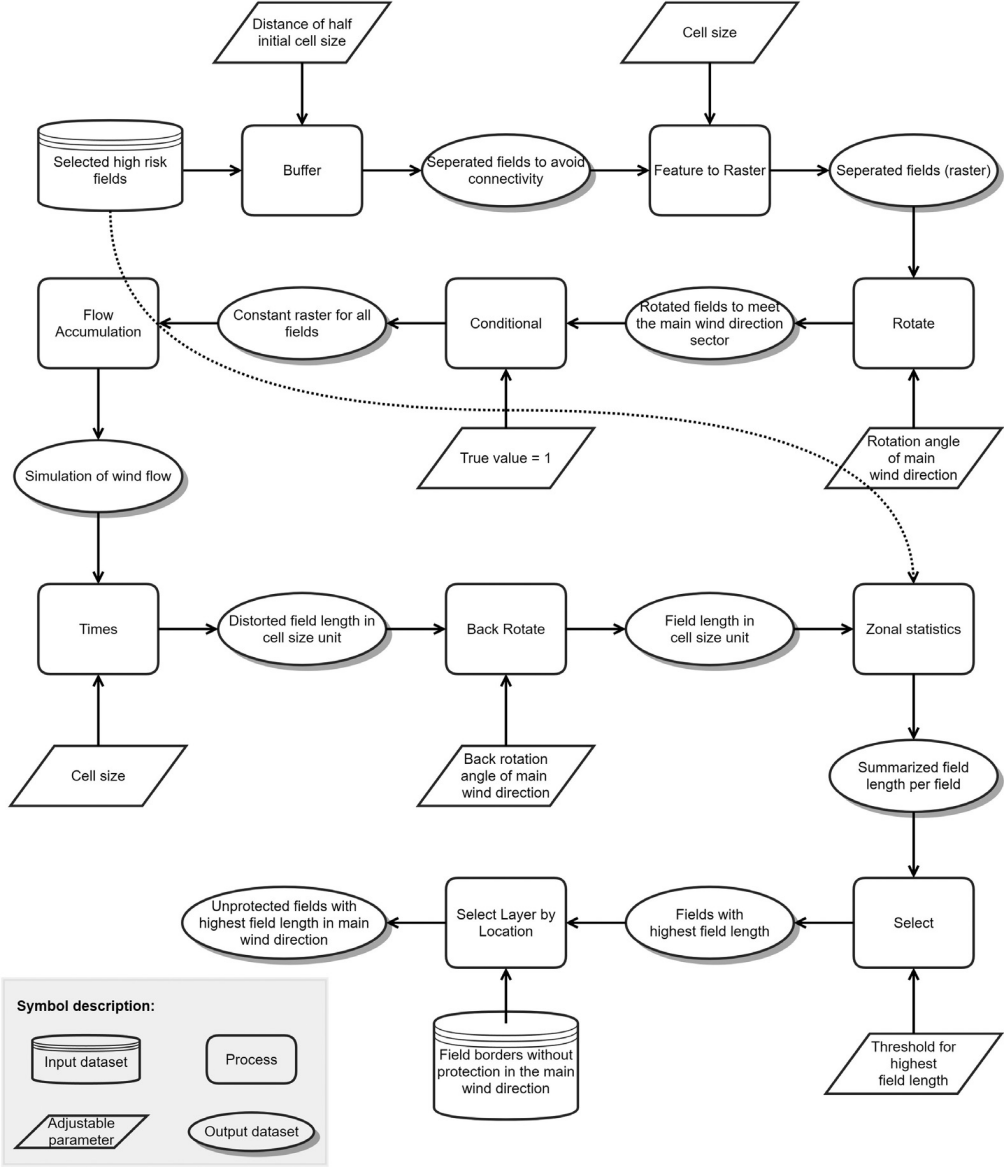
Model structure of module 4 to select unprotected field borders perpendicular to the main wind direction that belong to a portion of 10% of fields with the highest length.

The field length is an essential parameter in wind erosion studies as it indicates the distance a wind stream can overpass a field without any interruption by obstacles like windbreaks. As such, the wind stream and the transport capacity of wind forces on the topsoil can increase [16]. The process of accumulating sediments by overpassing a field is also called “avalanching effect” [17]. The field length in the main wind directions is calculated following Schmidt et al. [13], where the field length was calculated for multidirectional winds. The high risk fields are internally buffered by slightly more than a half cell size to allow a clear separation of individual fields from adjacent fields, converted to a grid format, and rotated to fit perfectly in the main working direction of the subsequent flow accumulation tool which is from left to right (Fig. 4). Prior to the flow accumulation, a constant value of 1 (direction left to right/West to East) to all in-field raster cells is introduced. The flow accumulation process results in an accumulation of connected cells until an interruption due to a field border is reached. As such, the process avoids a connectivity of field blocks. The accumulated values are multiplied by the cell size to result in the flow length in metric units. A back-rotation transforms the data to its original orientation. By using zonal statistics, each field is assigned by a certain aggregated field length. Setting a threshold value enables a comparison of single fields even within aggregated field blocks. Fields with high values have long field lengths, fields with low values are short in the main wind direction. The field length is further used in the next module as a suitability criteria for potential windbreak installation.

For the case study, we selected a threshold of the 90^th^-percentile to accumulate the field length values. To limit the fields that are most prone to wind erosion, we selected a portion of fields (10%) that have the highest field lengths (Fig. S4).

### Module 5: definition of criteria

**Fig. 5:**
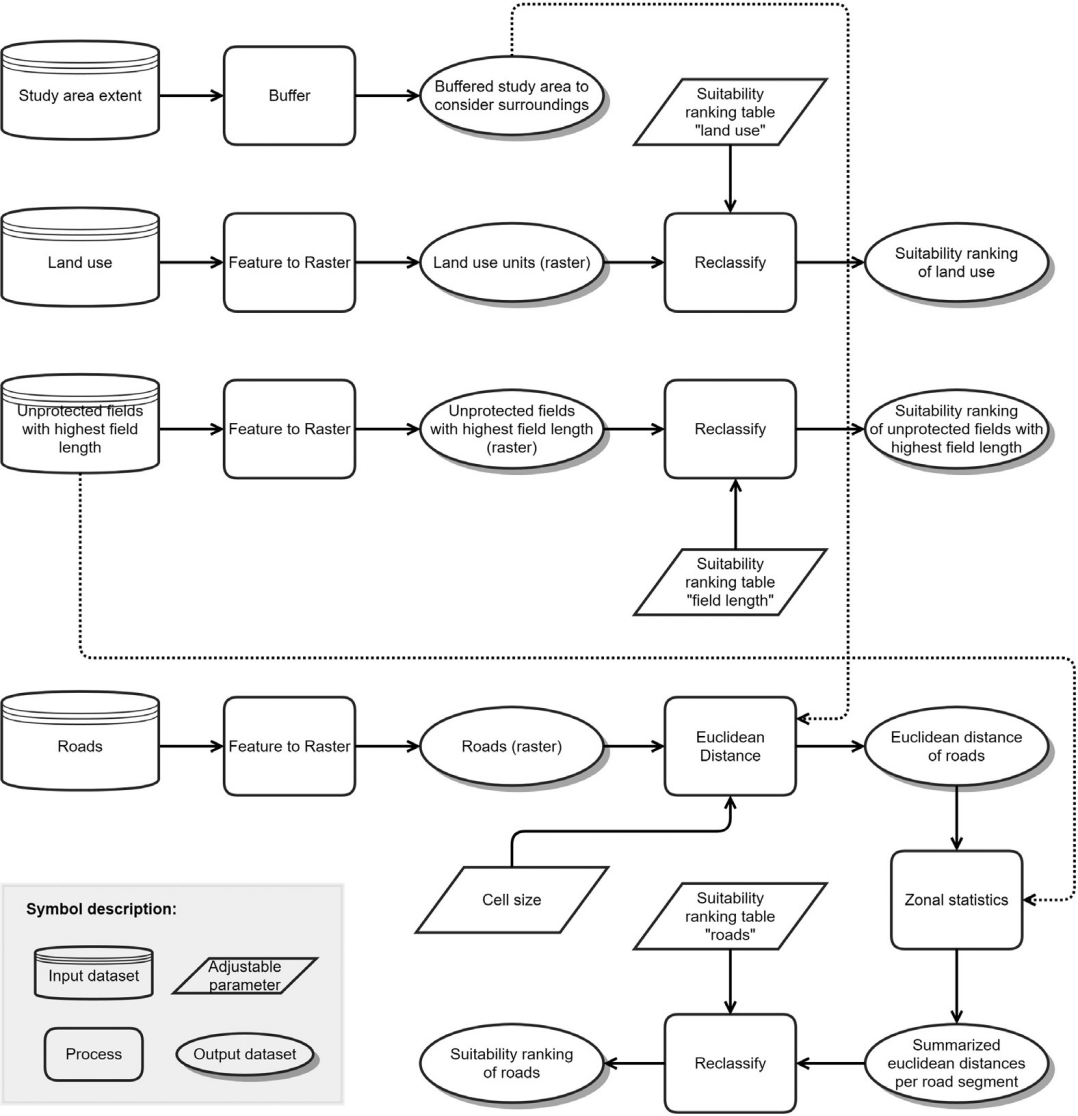
Model structure of module 5 to define the suitability ranking criteria.

Unlike the previous modules, module 5 (Fig. 5) does not select and thus limit the potential locations for windbreaks but defines criteria that evaluate the usefulness of planting new windbreaks at the remaining fields of module 4. The definition of criteria is crucial for setting up the specific demands of the user. This is the most variable module of TASOW as various settings of suitability levels control the model output.

Criteria 1 deals with the land use patterns as it is only possible to use certain land use types for introducing new windbreaks. Setting new windbreaks at an area which is currently under commercial or industrial use is somewhat unlikely while new windbreaks can more easily be installed on farmlands or grasslands. The best suitability is ranked high (10), and the lowest suitability is ranked low (1). The ranking can be altered by the user to be adjusted for specific needs.

Criteria 2 assumes that windbreaks closer to roads are preferable for two reasons: First, roads often border field blocks, which are hydrological units of continuous agricultural land and many fields [18] and thus represent the outermost boundary of aggregated fields. Second, roads are often affected by the off-site effects of wind erosion [19] and can be protected by roadside windbreaks. The distance attributed to each road is calculated by Euclidean distance and the classification is organized by an averaging of distances to each road segment. The distance classes as well as the suitability rank is suitable to the user needs and experience. Like for criteria 1, a ranking from 1 to 10 is chosen. The user must pay attention to the correct order of the ranks where high suitability equals 10 (close to roads) and low suitability equals 1 (distant to roads).

Criteria 3 considers the length of field borders. The longer the field border, the more effective the potential windbreak would be, since it would protect a field that has a large extension perpendicular to the wind direction. The classification is applied via quantiles of all field lengths.

Table 2 shows the ranking of land use types that are eligible for a transition to new plantations of windbreaks in the demonstration area.

**Table 2:** Suitability ranking of land use classes for a potential transition to windbreaks in the demonstration area. Higher ranks express higher suitability.

land use category in case study	code	suitability rank in case study
farmland	17	10
forest	1	10
orchard	20	10
scrub	11	10
vineyard	15	10
grass	4	9
meadow	10	9
nature reserve	18	8
park	2	8
cemetery	3	5
farmyard	14	5
military	7	5
other	21	5
recreation ground	6	5
allotments	9	1
commercial	5	1
heath	19	1
industrial	8	1
quarry	16	1
residential	13	1
retail	12	1

The suitability ranking for potential windbreak sites in the demonstration region in Austria as a function of distance from roads as well as for field lengths can be seen in Tables 3 and 4. Here, we choose the following distance classes for our demonstration area according to quantile classification method of 10 classes and assigned to subsequent suitability ranks.

**Table 3:** Suitability ranking of the potential windbreak locations in relation to the distance from roads in the case study area. The closer the potential site is to a road, the higher the ranking.

distance to roads (m) in case study		suitability rank in case study
from	to	
0	2	10
>2	6	9
>6	22	8
>22	42	7
>42	62	6
>62	78	5
>78	95	4
>95	123	3
>123	157	2
>157	507	1

**Table 4:** Suitability ranking of the field borders as an indicator for a more protective function of a potential windbreak in the demonstration region.

length of field borders (m) in case study		suitability rank in case study
from	to	
0	39	1
>39	56	2
>56	78	3
>78	105	4
>105	140	5
>140	192	6
>192	258	7
>258	350	8
>350	477	9
>477	1148	10

### Module 6: weighted overlay and final location selection

**Fig. 6:**
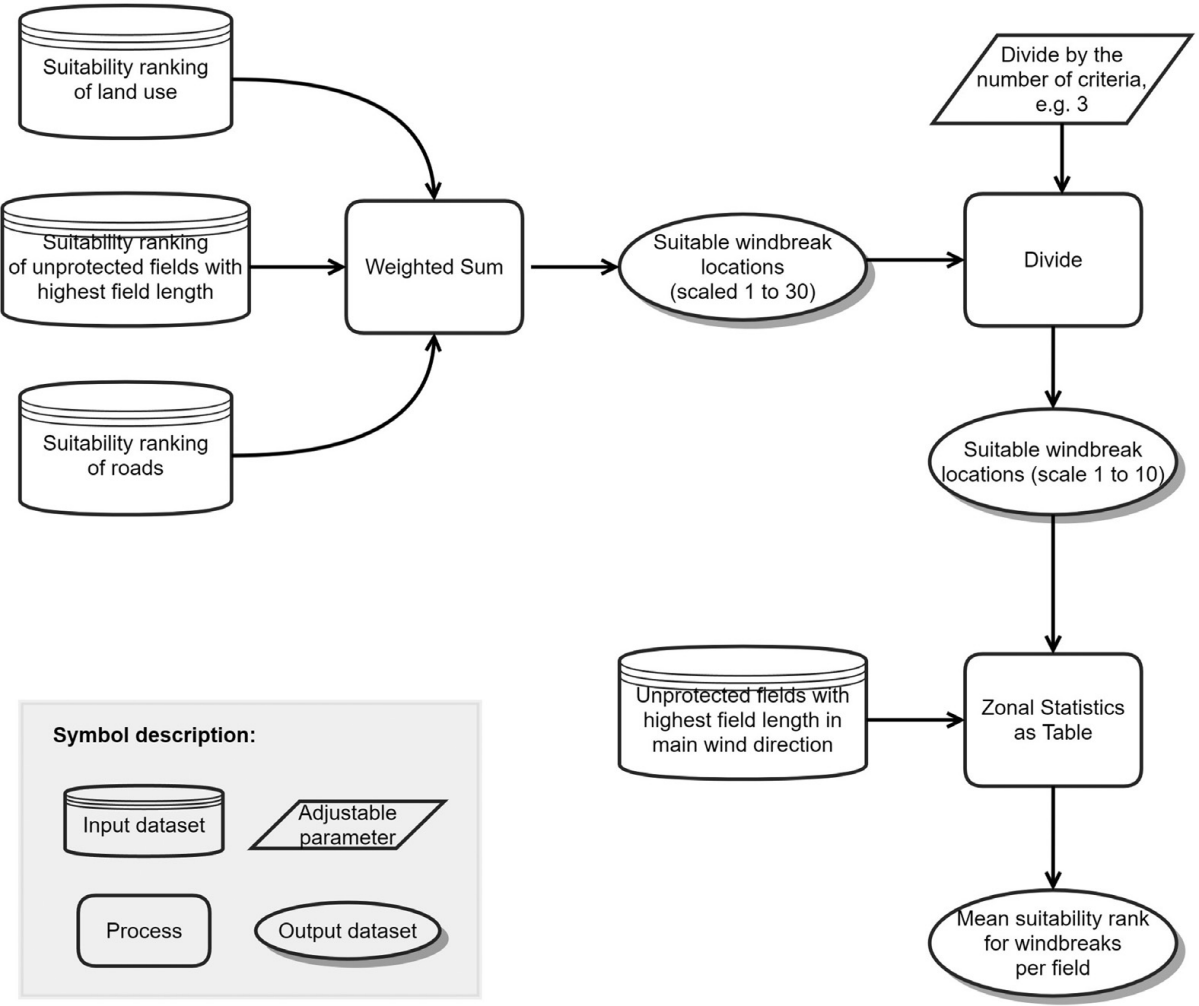
Model structure of module 6 to define the final potential locations for the planting of windbreaks under consideration of the criteria in module 5.

All three previously introduced criteria are weighted and overlaid (Fig. 6). The resulting raster-based weighting map defines potential locations for windbreaks. However, the dataset information is converted to a vector file (by zonal statistics and join) to preserve the original geometry of field borders relevant to the implementation of potential windbreak. Suitability scores range from 1 (very low suitability) to 10 (very high suitability).

The weighting of the criteria is subject to the user. The authors suggest an equal weighting of 33.3% as it was also used for the case study (Fig. S5) and ensured a uniform consideration of each criterion and avoids leveraging of specific criteria.

## Method validation

Potential windbreak sites are validated by a visual inspection based on high-resolution orthophotos (30 cm) from Geoland [14]. We selected 10% (n=332) of the 3320 suitable locations as control sites for a virtual inspection and verification. Visible inspection included i) the correct location of the potential windbreak on a field border and ii) any potential conflicts with existing windbreaks. The randomly selected locations are constrained not to interfere with each other, with a minimal spacing of 100 m.

**Fig. 7:**
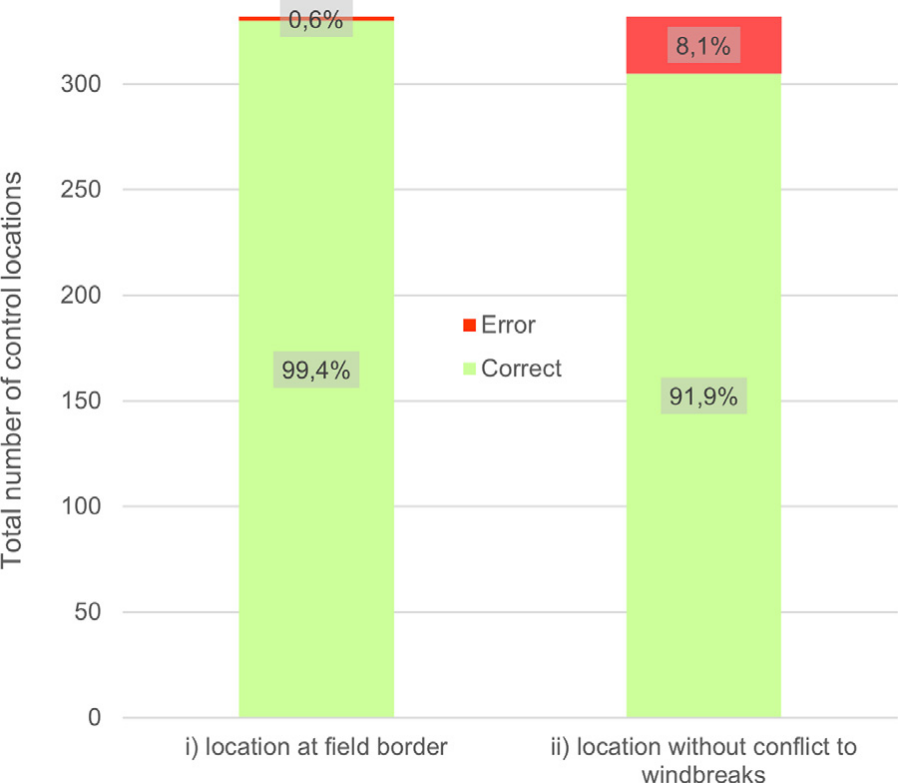
Error rates of visual inspection of the control locations (n=332).

For our study area in eastern Austria, there was a misclassification of 0.6% for the location of suitable windbreaks at field boundaries (Fig. 7). In general, the model workflow can select field boundaries and thus suitable windbreak locations at field boundaries very well.

Further validation results showed that 92% of all potential windbreak locations are correctly assessed when there are no already existing windbreaks (Fig. 7). In some cases, windbreak-like obstacles such as spaced tree rows or property hedgerows are present at suitable locations. However, this fact is mainly due to the definition and survey of windbreaks and/or the setting of the buffer distance in module 3 which controls the inclusion of existing windbreaks. A value of 10 m was chosen as parameter in the method as default which means that only very close windbreaks are considered here. The validation dataset is provided in the supplementary material.

The input RWEQ model, as well as its validity are already discussed in Scheper et al. [3] and are not part of the present work as it would not evaluate the methods validity but the input data.

Please note that we do not intend to provide an actual recommendation for planting windbreaks, but only to provide a planning tool and advise on suitable locations according to our automated routine. Since visual inspection of potential locations is mandatory anyway, an 8% margin of error is acceptable. Further policy and conservation investigations needs to be done to decide on an actual planting depending on land availability, biodiversity, land use regulations, etc.

Furthermore, it needs to be mentioned that vegetated windbreaks are one of multiple options to reduce wind erosion risk on agricultural soils. The highest effectivity will be gained by combinations of landscape management measures such as windbreaks and soil management or agronomical measures. Additionally, especially in arid zones, tree growth is often very slow or even impossible. Therefore, TASOW is considered to be especially suitable for use in temperate climates.

## Ethics statements

No ethical statements must be declared.

**Supplementary material and/or additional information**

The here presented model TASOW for the study region in Austria as well as the input data for the study region and the validation dataset are included as supplementary material and can be used in ESRI ArcGIS and altered according to the needs of the user.

## CRediT authorship contribution statement

**Simon Scheper:** Conceptualization, Methodology, Validation, Formal analysis, Investigation, Resources, Data curation, Writing – original draft, Writing – review & editing, Visualization. **Barbara Kitzler:** Writing – original draft, Writing – review & editing, Supervision. **Thomas Weninger:** Writing – original draft, Writing – review & editing. **Peter Strauss:** Writing – original draft, Writing – review & editing. **Kerstin Michel:** Conceptualization, Validation, Writing – original draft, Writing – review & editing, Supervision, Project administration, Funding acquisition.

## Declaration of Competing Interests

The authors declare that they have no known competing financial interests or personal relationships that could have appeared to influence the work reported in this paper.

## Data Availability

The here presented model TASOW for the study region in Austria as well as the input data for the study region and the validation dataset are included as supplementary material.

## Appendix Supplementary materials

Image, application 1
