# Supplementary material for: TASOW – A tool for the automated selection of potential windbreaks
Source: MethodsX. 2022 Aug 24;9:101826. doi: 10.1016/j.mex.2022.101826 (PMC9450165; doi:10.1016/j.mex.2022.101826)
Supplement: Supplementary file 1 [file mmc1.docx]

**Supplement Material**


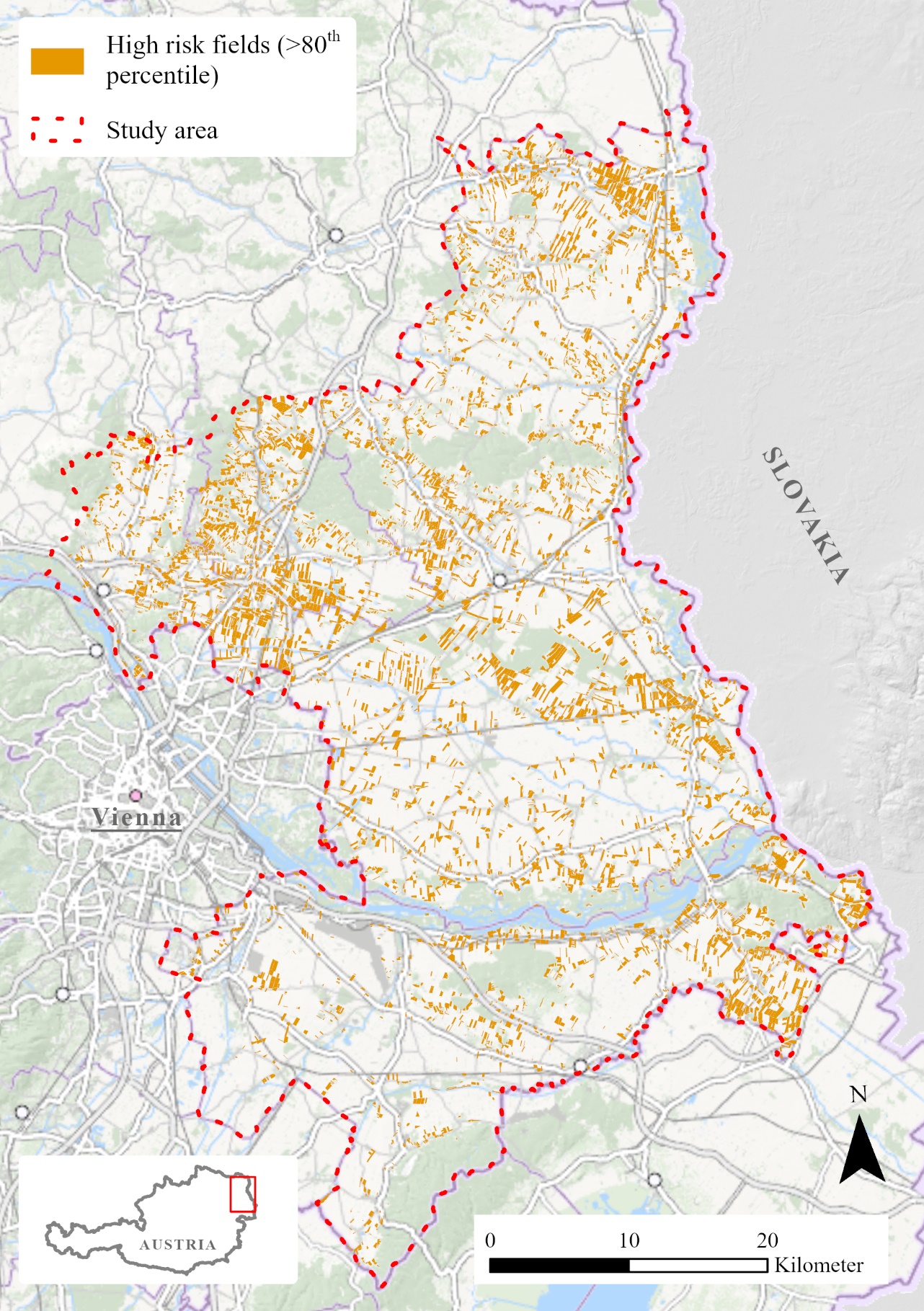


Figure S1: High risk fields due to wind erosion within the area of the demonstration study.


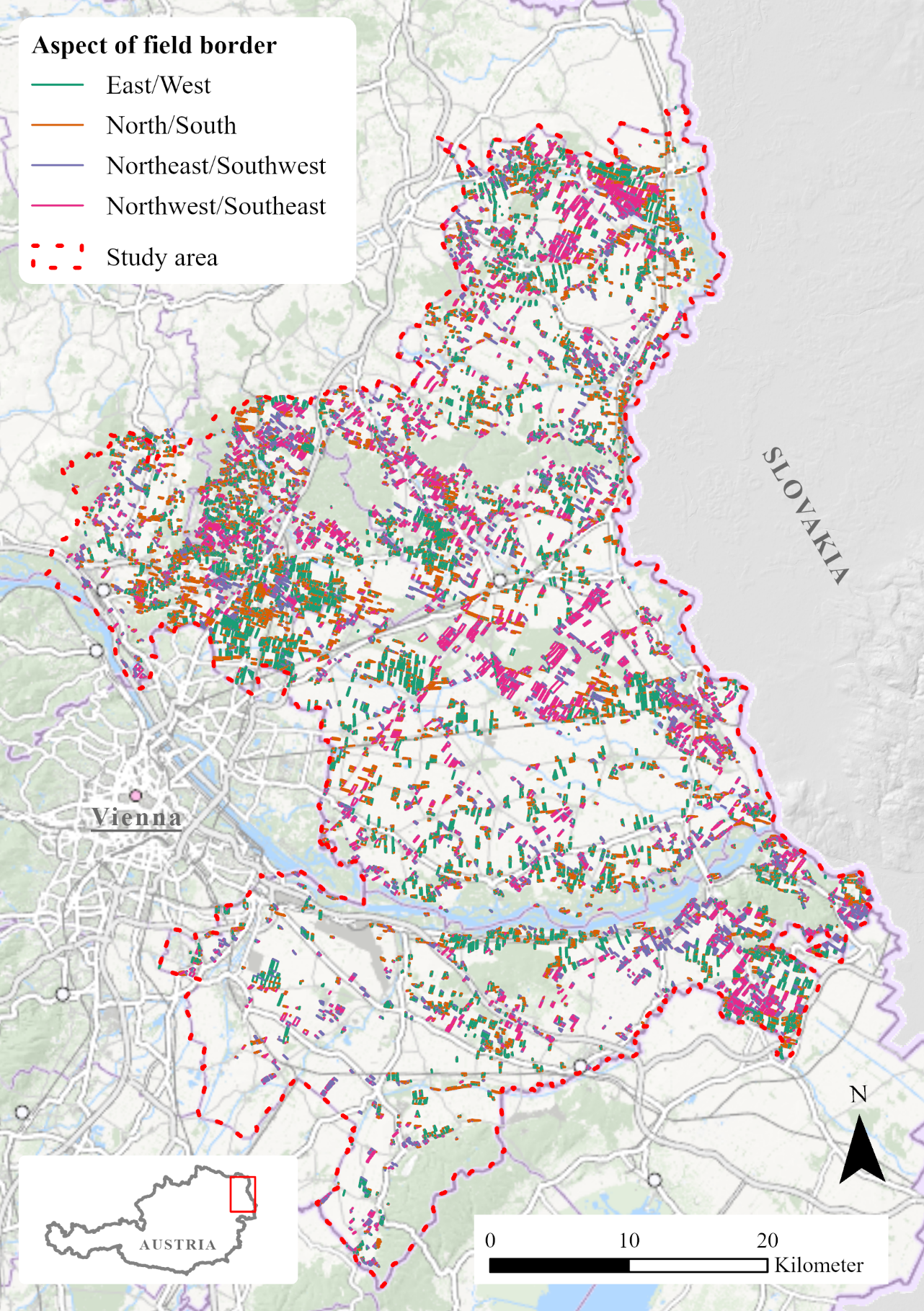


Figure S2: Aspect of field borders in the demonstration area according to the eight main wind direction classes (opposite directions are lumped together) of the fields with highest soil erosion risk.


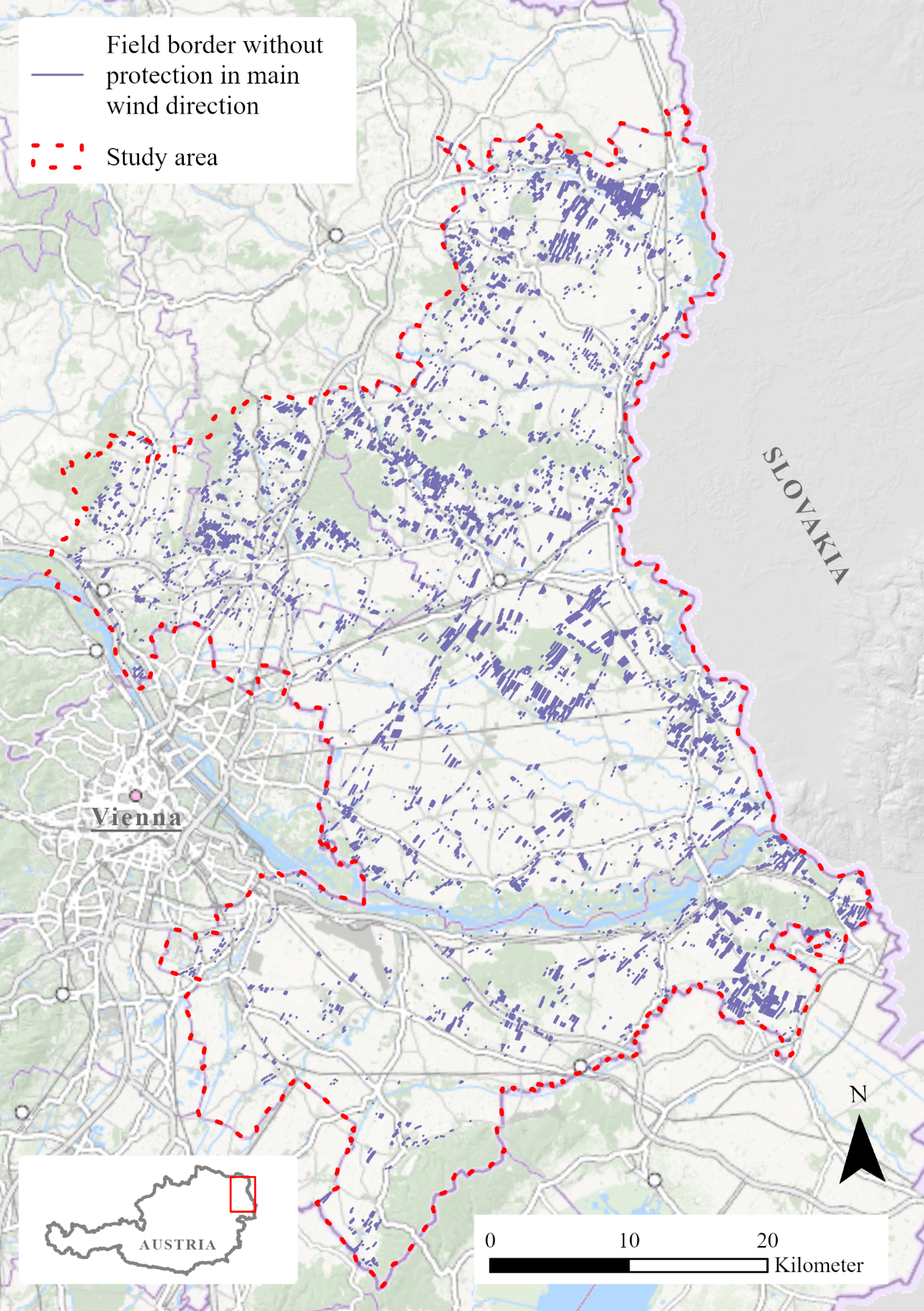


Figure S3: Remaining field borders of high risk fields not accompanied by vegetated windbreaks in the main wind direction


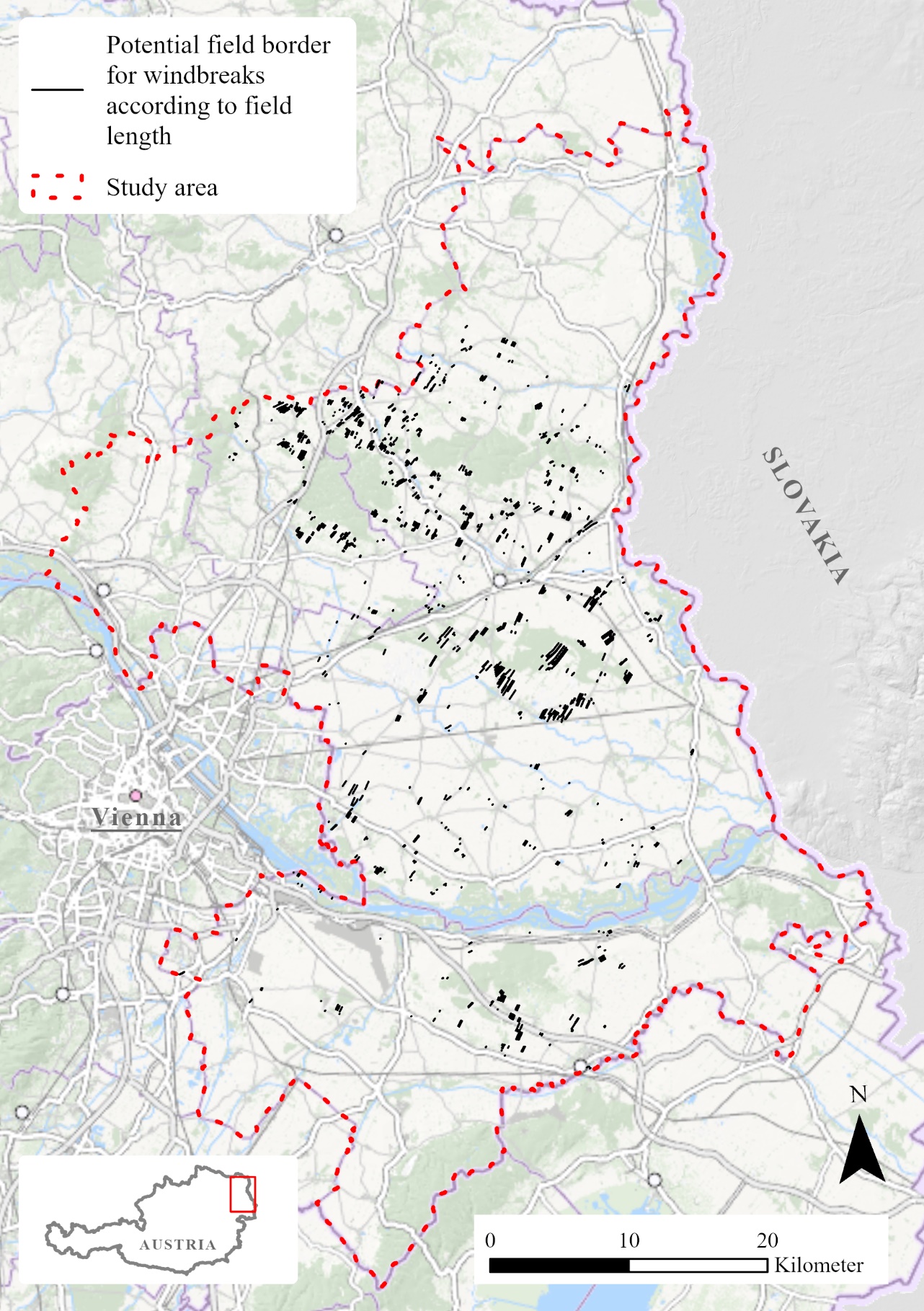


Figure S4: Unprotected field borders in aspect to the main wind direction that belong to the upper 10% of fields with highest field length.


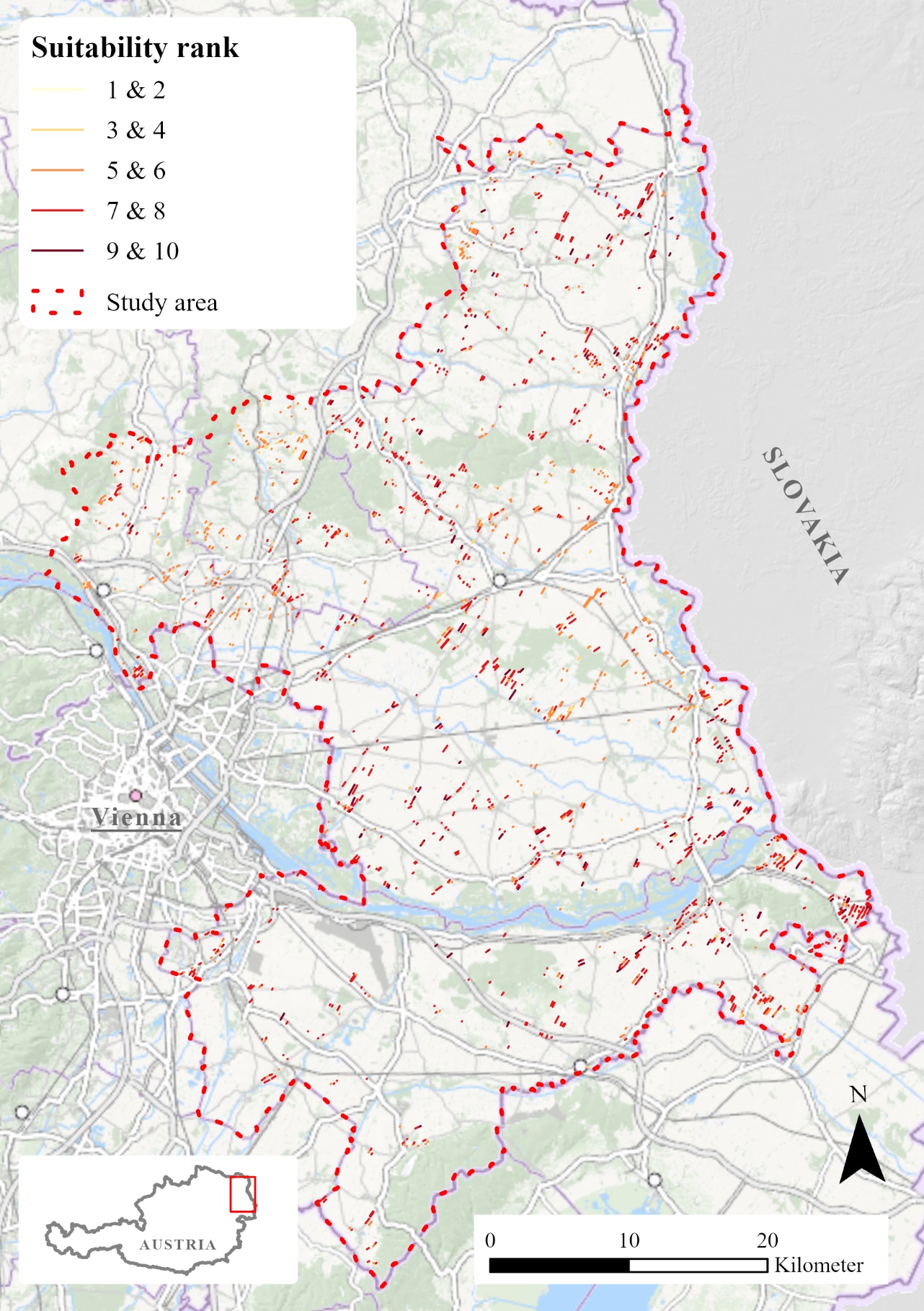


Figure S5: Potential sites for installation of windbreaks according to the soil erosion risk by wind and the defined criteria.

**Datasets and model**

Due to the file size the datasets and the model for the review process can be downloaded via the following link:

<https://drive.google.com/file/d/1qBJo-TwOPbGWBo-fMgMp1d5c38hEtxfb/view?usp=sharing>
